# Performance, heat tolerance response, and blood metabolites of water-restricted Xhosa goats supplemented with vitamin C^1^

**DOI:** 10.1093/tas/txaa044

**Published:** 2020-04-10

**Authors:** Oluwakamisi F Akinmoladun, Fabian N Fon, Conference T Mpendulo, Omobola Okoh

**Affiliations:** 1 Department of Livestock and Pasture Science, Faculty of Science and Agriculture, University of Fort Hare, Alice, Eastern Cape, South Africa; 2 Department of Animal and Environmental Biology, Faculty of Science, Adekunle Ajasin University, Akungba-Akoko, Ondo State, Nigeria; 3 Department of Agriculture, University of Zululand, Kwadlangezwa, Kwazulu-Natal, South Africa; 4 Department of Chemistry, Faculty of Science and Agriculture, University of Fort Hare, Alice, Eastern Cape, South Africa

**Keywords:** blood metabolites, thermotolerance, vitamin C, water stress, Xhosa ear-lobe

## Abstract

Water restriction in small ruminants is usually accompanied by a drop in feed intake, body weight, and disturbances in the normal internal milieu. However, attempts to lessen the burden of water stress with vitamin C (VC) supplementation have been greeted with conflicting reports. Therefore, this experiment was conducted to evaluate the effect of single and/or multiple VC supplementations in water-restricted Xhosa goats by evaluating their performance, heat tolerance, and blood metabolites. In total, 42 does, 12 mo old and with an average weight of 15.92 ± 2.12 kg were evaluated for 75 d. The does were distributed according to a complete randomized design into seven groups of six comparable animals: W0, without water restriction (control); W70, water restriction of 70% of ad libitum water intake (WI); W50, water restriction of 50% ad libitum WI; W70^+^, water restriction of 70% of ad libitum WI plus 3 g VC daily; W50^+^, water restriction 50% of ad libitum WI plus 3 g VC daily; W70^++^, water restriction of 70% of ad libitum WI plus 3 g VC and extra 5 g VC given every eighth day; and W50^++^, water restriction of 50% of ad libitum WI plus 3 g VC and extra 5 g VC given every eighth day. Goats under the W50 group were the most affected (*P* < 0.05) and the effect was more pronounced in their body condition scores (BCs). Weight loss due to water restriction was reduced by VC supplementation in treated groups. Changes in body thermal gradient, rectal temperature, cholesterol, and bilirubin were similar (*P* > 0.05) across the various experimental groups. The attenuation effect of VC was significant (*P* < 0.05) in responses to respiratory rate, Na^+^, K^+^, Mg^2+^, Cl^−^, Ca^2+^, and urea. Supplementation of VC (either single or multiple) did not significantly (*P* > 0.05) improve the effect of water restriction on BCs, FAMACHA, glucose, globulin, alanine aminotransferase, and high-density lipoprotein. The additive effect of multiple VC significantly influenced (*P* < 0.05) Na^+^ and Mg^2+^. Limited WI affects growth and other physiological parameters in Xhosa goats. However, supplementation of VC may be beneficial at modulating the stressful stimuli imposed by water stress.

## INTRODUCTION

Small livestock, such as goats, contributes immensely to the reduction of poverty and improvement in livelihoods, especially for resource-limited rural communities and marginalized families in dry and water-limited regions of the world. The pivotal role they play ranges from the provision of animal protein, income generation from sales either as live animals or their primal cuts in the markets, to religious and/or cultural purposes. However, the sustainable productions of livestock are under threat due to increasing water scarcity and fluctuating precipitation. Irregularities in rainfall patterns caused by global warming and vagaries in weather conditions have limited the amount of fresh water available to most regions around the world (Kurylyk and MacQuarrie, 2013). South Africa is considered a water-scarce country (Donnenfeld et al., 2018) and goat production systems in the semiarid region of the Eastern Cape Province range from nomadic to the semisedentary or exclusive scavenging type. Under this traditional pastoral farming system, animals are forced to walk a long distance in search of water and feed throughout the year. Usually, the watering points accessible to these animals cannot be relied upon because they get dried up easily during the dry summer season. Animals in the field are, therefore, faced with dehydration while grazing far from widely spread watering points in order to meet their nutrient requirements.

Goats, especially the indigenous ones, are more adaptable to harsh environmental conditions of drought and heat (Silanikove, 2000). They can efficiently utilize limited forage and are less susceptible to endemic diseases as compared to exotic or nonadaptable breeds. The Xhosa ear-lobe breed, indigenous to the Eastern Cape region, is well adapted to the semiarid environment characterized by temperature extremes and limited water availability, a trait acquired through natural selection over the years. However, thermotolerance and ability to withstand suboptimal intake of water vary with animal types, breed, and extent of adaptability. Although adaptation can be enhanced following prolonged exposure by livestock, studies have documented huge differences in response in the different breeds (Habibu et al., 2017). For example, desert goats raised under traditional systems may be watered once every 3–6 d during water scarcity, while the Black Bedouin and Barmer goats, an adapted breed, can live on a once every 4 d watering regime (Silanikove, 1994). A comprehensive review of the adaptability and tolerance of small ruminants and their breeds to different levels of water deprivation and/or restriction has been documented (Akinmoladun et al., 2019). Despite this efficient water use in adaptable breeds, disturbances of water balance portend a stressful stimulus and have been shown to hamper feed intake and body weight and influence the body heat balance, as well as the composition of the body fluids (Akinmoladun et al., 2019).

Supplementation of vitamin C (VC) to the diets of ruminants is not a common practice because they can biosynthesize L-ascorbic acid through the glucuronic acid pathway in the liver and kidney (McDowell, 2000). However, plasma VC is usually depleted during stress and disease conditions (Ranjan et al., 2005), hence a possibility of improvement if supplemented. Despite the positive outcome following VC supplementation, others have reported a nonsignificant effect. For example, oral VC (ascorbic acid) supplementation lessens body weight loss in goats exposed to heat and transportation stress (Minka and Ayo, 2012). Also, the ability of ewes to withstand limited water intake (WI; Ghanem et al., 2008), reproductive traits of pregnant ewes, and the weight of their new-born lambs were enhanced following VC supplementation (Haliloglu and Serpek, 2000). However, VC supplementation was found to have little or no effect on summer heat-induced stress in Rahmani ewes (Hashem et al., 2016). Also, the results obtained following supplementation of transportation-stressed Holstein heifers with dietary ascorbyl-2-phosphate were indecisive (Tyler and Cummins, 2003).

The mechanisms that allow this indigenous goat breed to thrive, despite the hard and unfavorable environmental conditions are yet to be investigated. Furthermore, information about the response of Xhosa ear-lobe goats, following supplementation of different doses of VC, to the combined effect of water stress and high ambient temperature is scarce. It was hypothesized that bioavailability of VC could be boosted following multiple VC supplementations and, ultimately, help to reduce the burden of water stress imposed on the animals. Therefore, this study focused on growth performance, heat tolerance, and blood metabolites of water-restricted Xhosa ear-lobe supplemented with VC.

## MATERIALS AND METHODS

All experimental procedures complied with the guidelines of the Research Ethics Committee of the University of Fort Hare, South Africa (Ref. No: MUC011SAKI01).

### Study Site Description

The experiment was conducted at the Honeydale farm, University of Fort Hare. It is located 5 km east of the town of Alice, Eastern Cape, South Africa, and is 520 m above sea level. It is situated in the False Thornveld of the Eastern Cape with geographical coordinates 32^o^ 46^′^S and 26^o^ 52^′^E and receives 480–490 mm annual rainfall. The study was conducted during the summer season.

### Experimental Animals and Management

Forty-two female goats (Xhosa breed) with an average age of 12 months and body weight 15.92 ± 2.12 kg were used for the 75-d trials. The goats were dewormed using ivermectin and vaccinated against the foot-and-mouth disease before the experiment. They were kept in individual metabolic cages (1.33 × 0.58 m), provided with feeder and a water trough. The animals were weighed at the beginning of the trial and feed was offered as total mixed ration (TMR) based on 4% of their body weights in the ratio of 70:30 of Lucerne hay and concentrate on dry matter (DM) basis, respectively. The ingredient and nutrient composition of the experimental diet is shown in Table 1.

**Table 1.** Composition of experimental diet (g/kg as fed)

Ingredient	Quantity
Lucerne	700
Maize gluten	166.3
Sunflower husk	127.3
Limestone	2.1
MCDP	2.3
Salt	1.5
Premix^a^	0.6
Calculated composition	
Organic matter	889.5
Crude protein	216.7
Ether extract	17.5
Crude fiber	215.3
Nitrogen free extract	440
Phosphorous	3.8
Calcium	15.7
Magnesium	6.0
Potassium	8.3
Sodium mg/kg	2263
Copper mg/kg	41
Iron mg/kg	116
Manganese mg/kg	38
Zinc mg/kg	18

MCDP, monosodium diphosphate.

^a^Ca = 220 g/kg; P = 55 g/kg; Mg = 35g/kg; S = 22 g/kg; Cl = 105g/kg; Na = 70 g/kg; Mn = 1,500 mg/kg; Fe = 500 mg/kg; Zn = 1,550 mg/kg; Cu = 440 mg/kg; Co = 50 mg/kg; I = 40 mg/kg; Se = 20 mg/kg.

### Dietary Treatments and Design

The animals were balanced for body weight and randomly assigned to the seven dietary treatment groups: W0, without water restriction (control); W70, water restriction of 70% of ad libitum WI; W50, water restriction of 50% ad libitum WI; W70^+^, water restriction of 70% of ad libitum WI plus 3 g VC daily; W50^+^, water restriction 50% of ad libitum WI plus 3 g VC daily; W70^++^, water restriction of 70% of ad libitum WI plus 3 g VC and extra 5 g VC given every eighth day; W50^++^, water restriction of 50% of ad libitum WI plus 3 g VC and extra 5 g VC given every eighth day. It was assumed that the control group (W0) would not be stressed due to its unhindered access to ad libitum WI and, thus, exempted from VC supplementation. The L-ascorbic acid (VC) used as a supplement was sourced from Minema Chemical Stores, Gauteng, South Africa. All animals receiving VC supplementation were subjected to a 6-d preparation period (coinciding with the last 6 d during adaptation period) during which they were orally supplemented with 10 g VC/50 mL water/animal. This was based on a previous work by Ghanem et al. (2008) to make sure that the animals were at the same VC status. The 3 g/d VC dose was selected based on previous findings on the effectiveness and higher bioavailability at lower doses (Tyler and Cummins, 2003). Also, multiple dosing of oral VC supplementation was reported to be more effective in boosting VC levels as compared to single dosing (Hidiroglou *et al.*, 1997). The experimental trial started in January and lasted for 89 consecutive days. The trial was preceded by a 14-d preliminary period.

### Feed and Growth Measurements

Feed was offered two times a day, at 0900 and 1600 hours in equal proportions. The weight of the TMR offered and refused was recorded daily to derive feed intake. Animals were weighed every 7 d before morning feeding and the average daily gain (ADG) calculated by dividing final body weight with days of trial. Water restriction percentages for experimental groups were calculated based on daily ad libitum intake of the control (W0) group. Water was supplied in containers of known volume and was topped-up once a day. In the control (W0) group, does receive ad libitum water daily at two different times of the day at 0800 and 1500 hours to determine the quantity of water ingested. Total WI was calculated as the difference between the amounts offered and leftovers, rebating loss of water due to evaporation. Water loss due to evaporation was calculated by putting buckets filled with water at focal points in the pen so that loss due to evaporation can be inputted when calculating for total WI. W70 and W50 groups did receive drinking water daily at a level of 70% and 50% of the total WI recorded in the control group, respectively. The efficiency of water use was determined by finding the ratio of WI to DM intake (WI:DMI).

### FAMACHA and Body Condition Scores

Body condition score (BCs) was determined by palpating the spinous processes of the lumbar vertebrae and assigning a score as follows: 1 (very lean, sharp prominent backbone and spinal processes and little flesh coverage); 2 (lean); 3 (medium, slight rounding of flesh over spine); 4 (fat); and 5 (very fat, cannot detect any backbone or spinal processes) at 0.5 increments (McGregor, 2011). Goats were also monitored for evidence of anemia by checking the color of the ocular mucous membranes using the FAMACHA eye chart and score assigned as follows: 1 (optimal; red color nonanemic); 2 (acceptable; red-pink color nonanemic); 3 (borderline; pink mildly anemic); 4 (dangerous; pink-white anemic); and 5 (fatal; porcelain-white; severely anemic; Kaplan et al. 2004).

### Meteorological Parameters and Heat Tolerance Measurements

Ambient temperature and relative humidity were recorded hourly in the experimental station with a portable data logger (Model: MT669, Major Tech, South Africa) during the 75-d trial. Temperature-humidity index (THI) was calculated for the whole 75 d of the treatment. The 75 d were divided into five intervals of 15 d each and THI was similarly calculated for each of these intervals. The equation described by Marai et al. (2007), in which; THI = db °C − [(0.31 − 0.31 RH %) (db °C − 14.4)], where db °C = dry bulb thermometer in Celsius and RH = relative humidity (%) was used. The extent of heat stress was determined based on the THI values (i.e., THI <22.2 = absence of heat stress; 22.2 to <23.3 = moderate heat stress; 23.3 to <25.6 = severe heat stress; and 25.6> = extreme severe heat stress; Marai et al., 2007). Rectal temperature (RcT, °C) and respiration rate (RR, breathes/min) were recorded between 0900 and 1400 hours on days 15, 30, 45, 60, and 75 of the treatment period. RcT (°C) was measured after inserting a clinical digital thermometer about 5 cm deep into the rectum of animals and making contact with the mucous membrane for 2 min. RR (breaths/min) was obtained through the counting of flank movements during 1 min with each inward–outward flank movements counted as one complete respiration. Skin temperatures on the neck, belly, and thurl regions were measured using an infrared thermometer (Nubee NUB8380 Temperature Gun, California, USA). The thermometer was held at a 15-cm distance away from the animal without direct contact. The average skin temperature of each goat in the current study was calculated by averaging the temperature on the neck, belly, and thurl region. Body thermal gradients were calculated according to Richards (1973) using the formulas: Internal gradient = RcT − skin temperature; External gradient = RcT − ambient temperature; Total thermal gradient = skin temperature − ambient temperature.

### Blood Sampling

Blood samples were collected from the jugular vein of each goat (three animals per treatment) into ethylene diamine tetraacetic acid-coated and heparinized vacutainer tubes with BD Hemogard on days 30, 60, and 75. Blood samples in heparinized tubes were centrifuged for 10 min at 3,500 rpm using a Model 5403 centrifuge (Geratebay Eppendorf GmbH, Engelsdorp, Germany). Thereafter, the centrifuged samples were stored at −20 °C until analysis was conducted. The obtained plasma samples were analyzed using a Checks machine (Next/Vetex Alfa Wasserman Analyser) and commercially purchased kits (Siemens). The concentrations of blood serum total protein, albumin, creatinine, and alkaline phosphate (ALP) were spectrophotometrically analyzed using colorimetric procedures. Globulin concentration was calculated by subtracting albumin values from the corresponding total protein values. Glucose, total cholesterol, and urea were analyzed using enzymatic methods, while aspartate transaminase and alanine transaminase were determined using ultraviolet techniques.

### Statistical Analysis

Analysis of variance was performed based on averages per treatment groups using the GLM procedure of SAS (2013) to determine the fixed effect of treatment, experimental period, their interactions, and the random effect of animals. Models: *Y*_*ijk*_ = µ + *T*_*i*_ + *D*_*j*_ + (*TD*)_*ij*_ + *ɛ*_*ijk*_ and *Y*_*ij*_ = *µ* + *T*_*i*_ + *ɛ*_*i*j_ for nonrepeated measures, in which *Y*_*ijk*_ is the value of the dependent variable determined from an observation taken from each animal, µ is the overall mean, *T*_*i*_ is the fixed effect of the *i*th treatment (*i* = 1:7), *D*_*j*_ is the fixed effect of the *j*th day of observation (*j* = 1:5), (*TD*)_*ij*_ is the interaction between treatment and day of observation and *ɛ*_*ijk*_ is the random error. Analyzed data were considered significant at *P* < 0.05.

## RESULTS

### THI, DMI, and body weight changes

**Figure 1.**
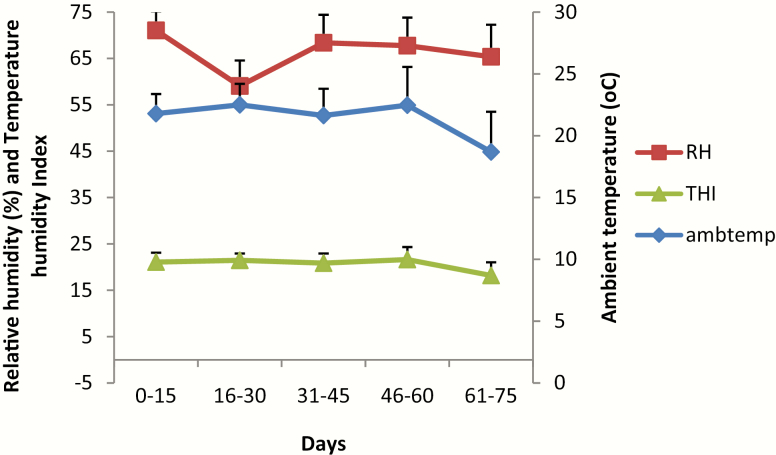
Diurnal temperature and humidity pattern in the housing unit.

Diurnal temperature (^o^C) and humidity (RH %) pattern in the housing units and body weight changes are shown in Fig. 1 and Table 2, respectively. The average THI in the experimental room was 25.57 ± 0.15 throughout the experiment. The final body weight after 75 d of water restriction across the various treatment groups did not differ (*P* > 0.05). However, values recorded for animals subjected to water restriction in groups W70 and W50 were low compared to the control (W0) group. This decrease in final weight was attenuated in the treated groups (W70^+^, W50^+^, W70^++^, and W50^++^). The extra dose of VC given to the animals on the eighth day (W70^++^ and W50^++^) did not further lessen the weight loss compared to those on single-dose (W70^+^ and W50^+^) groups. Water restriction levels had a significant effect (*P* < 0.05) on body weight gain and average weight gain. DMI was significantly higher (*P* < 0.05) in the control (W0) group compared to the entire water-restricted groups. The WI followed the watering regimen adopted and decreased (*P* < 0.05) as the percentage of ad libitum water given decreased.

**Table 2.** Performance of water-restricted Xhosa goats supplemented with VC

Parameter	W0	W70	W50	W70^+^	W50^+^	W70^++^	W50^++^	SEM	*P*-value
IW, kg	15.70	15.63	16.17	16.17	15.83	15.90	16.10	2.24	0.423
FW, kg	17.93	13.93	13.70	14.90	14.53	14.30	14.37	2.07	0.072
Gain, kg	2.23^a^	−1.70^bc^	−2.47^c^	−1.27^b^	−1.30^b^	−1.60^bc^	−1.73^bc^	0.37	0.021
ADG, g/d	29.78^a^	−22.67^bc^	−32.89^c^	−16.89^b^	−17.33^b^	−21.33^bc^	−23.11^bc^	4.87	0.015
BW^0.75	8.67	7.16	7.08	7.56	7.43	7.34	7.37	0.78	0.124
DMI, g/d	614.91^a^	369.09^b^	324.54^c^	376.21^b^	332.27^c^	376.03^b^	330.60^c^	7.76	0.017
WI, kg	92.47^a^	62.40^b^	45.61^c^	62.40^b^	45.61^c^	62.40^b^	45.61^c^	4.12	<0.0001
WI:DMI	2.31^a^	2.25^a^	1.87^b^	2.20^a^	1.83^b^	2.21^a^	1.84^b^	0.09	0.012

BW^0.75, metabolic weight; IW, Initial weight; FW, final weight; W, water restriction.

^a,b,c^Means with different superscripts across the row are significantly different (*P* < 0.05).

^+^3 g VC daily; ^++^3 g VC daily + extra 5 g VC every eighth day.

### Body Thermal Gradient and Physiological Response

The effect of water restriction with or without VC on body thermal gradient and physiological responses (respiratory rate [RR] and RcT) are shown in Tables 3 and 4. Water restriction levels did not significantly affect (*P* > 0.05) the skin temperature, internal gradient, external gradient, and total thermal gradient. The effect of water restriction levels with or without VC supplementation was not significant on the RcT (*P* > 0.05). All the water-restricted groups had significantly lower RR (*P* < 0.05) compared to the control (W0) group. The slight increase in the values recorded for RR in the VC-supplemented groups (W70^+^, W50^+^, W70^++^, and W50^++^) did not follow a consistent pattern compared to the untreated groups (W70 and W50).

**Table 3.** Body thermal gradients of water-restricted Xhosa goats supplemented with VC

		Treatment								Probability		
Parameter	Day	W0	W70	W50	W70^+^	W50^+^	W70^++^	W50^++^	SEM	T	D	T × D
Skin temperature, °C									0.322	ns	***	ns
	15	32.7	32.7	32.7	32.1	32.27	32.28	31.68				
	30	30.8	31.4	31.4	30.8	31.23	31.39	31.01				
	45	25.8	23.7	23.7	25.1	25.15	24.11	23.97				
	60	29.0	28.8	28.8	28.7	28.98	28.73	28.88				
	75	26.6	26.3	26.3	26.8	26.17	26.56	26.33				
Internal gradient									0.418	ns	***	ns
	15	6.33	6.17	6.57	6.99	6.20	6.45	6.79				
	30	7.76	7.11	7.30	7.19	7.20	7.44	7.56				
	45	12.43	14.16	13.52	12.08	12.85	13.66	14.17				
	60	8.20	9.65	9.30	9.73	9.52	8.34	9.39				
	75	11.81	11.53	11.43	12.09	11.25	11.44	11.87				
External gradient									0.272	ns	***	ns
	15	16.61	16.64	17.04	17.21	16.67	16.94	16.67				
	30	16.13	15.43	16.06	15.67	15.93	16.33	16.07				
	45	15.86	16.39	15.99	15.56	16.36	15.93	16.49				
	60	16.44	17.37	17.71	17.34	17.44	17.31	17.20				
	75	19.48	19.38	19.41	19.88	19.74	19.31	19.51				
Total gradient									0.322	*	***	ns
	15	10.28	10.47	10.47	10.22	10.47	10.49	9.89				
	30	8.38	8.54	8.76	8.48	8.73	8.89	8.51				
	45	3.42	2.22	2.47	3.47	3.50	2.46	2.33				
	60	8.24	7.72	8.41	7.61	7.92	8.97	7.82				
	75	7.67	7.84	7.98	7.79	8.49	7.86	7.64				

D, day effect; T, treatment effect; T × D, interaction between treatment and day; W, water restriction.

^abc^Means with different superscript down the column are significantly different (*P* < 0.05).

ns, *P* > 0.05; *P < 0.05; **P < 0.001; ***P < 0.0001.

^+^3 g VC daily; ^++^3 g VC daily + extra 5 g VC every eighth day.

**Table 4.** RcT and RR of water-restricted Xhosa goats supplemented with VC

		Treatments								Probability		
Parameter	Day	W0	W70	W50	W70^+^	W5^0+^	W70^++^	W50^++^	SEM	T	D	T × D
RcT, °C									0.27	ns	**	ns
	15	38.40	38.43	38.83	38.48	38.67	38.73	38.47				
	30	38.63	38.93	38.57	38.47	38.43	38.83	38.57				
	45	39.50	38.03	37.93	38.20	38.00	37.97	38.13				
	60	38.50	38.43	38.77	38.40	38.50	38.37	38.27				
	75	38.17	38.07	38.10	38.47	38.43	38.00	38.20				
RR, breathes/min									1.34	**	**	*
	15	38.67^a^	31.33^b^	30.53^b^	32.67^b^	31.17^b^	33.05^b^	31.05^b^				
	30	39.00^a^	32.33^bcd^	30.67^bc^	33.67^bc^	30.89^cd^	34.67^b^	30.79^d^				
	45	44.00^a^	35.33^b^	31.33^c^	35.43^b^	32.00^c^	36.67^b^	31.59^c^				
	60	42.67^a^	35.39^b^	33.33^b^	35.67^b^	33.67^b^	35.00^b^	33.52^b^				
	75	41.33^a^	34.51^b^	31.34^c^	36.67^b^	31.67^c^	35.67^b^	32.03^c^				

D, day effect; T, treatment effect; T × D, interaction between treatment and day; W, water restriction.

^abc^Means with different superscript down the column are significantly different (*P* < 0.05).

ns, *P* > 0.05;*P < 0.05; **P < 0.001; ***P < 0.0001.

^+^3 g VC daily; ^++^3 g VC daily + extra 5 g VC every eighth day.

### FAMACHA and BCs

The FAMACHA and BCs are shown in Table 5. Levels of water restriction, with or without VC did not significantly (*P* > 0.05) affect the FAMACHA scores. However, the BCs were significantly affected (*P* < 0.05) by levels of water restriction. VC-treated groups (W70^+^, W50^+^, W70^++^, and W50^++^) were not statistically different (*P* > 0.05) from the untreated groups (W70 and W50) in their BCs. The BCs of goats under W50 were the most affected (*P* < 0.05) compared to other water-restricted groups. All water-restricted groups had lower BCs compared to the water ad libitum groups.

**Table 5.** FAMACHA and BCs of water-restricted Xhosa goats supplemented with VC

		Treatments								Probability		
Parameter	Day	W0	W70	W50	W70^+^	W50^+^	W70^++^	W50^++^	SEM	T	D	T × D
FAMACHA									0.293	*	**	ns
	15	2.67^ab^	2.33^b^	2.33^b^	2.67^ab^	2.67^ab^	3.00^a^	2.33^b^				
	30	2.33^b^	2.33^b^	2.00^b^	2.33^b^	2.33^b^	2.33^b^	3.00^a^				
	45	3.00^ab^	2.67^b^	3.00^ab^	2.67^b^	3.00^ab^	3.00^ab^	3.33^a^				
	60	3.00	3.00	3.33	3.33	3.00	3.33	3.33				
	75	2.67	2.67	3.00	3.00	3.00	3.00	2.67				
BCs									0.252	***	*	ns
	15	3.00	2.33	2.33	2.67	2.67	2.33	2.67				
	30	3.33	2.33	2.00	2.33	2.00	2.67	2.33				
	45	2.67^a^	2.33^ab^	1.33^c^	2.33^ab^	2.33^ab^	2.33^ab^	2.00^bc^				
	60	3.00^a^	2.00^b^	2.00^b^	2.00^b^	2.00^b^	2.67^a^	2.00^b^				
	75	3.00^a^	2.67^ab^	2.00^c^	2.00^c^	2.00^c^	2.33^bc^	2.00^c^				

D, day effect; T, treatment effect; T × D, interaction between treatment and day; W, water restriction.

^abc^Means with different superscript down the column are significantly different (*P* < 0.05).

ns, *P* > 0.05; *P < 0.05; **P < 0.001; ***P < 0.0001.

^+^3 g VC daily; ^++^3 g VC daily + extra 5 g VC every eighth day.

### Blood Metabolites

The plasma osmolality and blood chemistry are shown in Tables 6 and 7. The effect of water restriction levels was significant on plasma osmolality. Plasma osmolality tends to increase (*P* < 0.05) with water restriction levels. However, the high plasma osmolality (Na, K, Mg, Cl, and Ca) due to water restriction levels were lowered following the administration of VC. The effect of extra VC dosing (W70^++^ and W50^++^) did not significantly (*P* > 0.05) influence the plasma osmolality compared to single dosing (W70^+^ and W50^+^). Supplementation of VC at both single and multiple doses had no significant effect (*P* > 0.05) on bilirubin, cholesterol, high-density lipoprotein and low-density lipoprotein (HDL and LDL). However, increased (*P* < 0.05) concentrations of urea, total protein, alanine aminotransferase (ALT), and ALP following water restriction levels were slightly attenuated in the VC-supplemented groups. Both single and multiple VC supplementations significantly influenced (*P* < 0.05) creatinine and glucose concentrations. Plasma triglyceride was significantly affected (*P* < 0.05) by water restriction levels and VC dosing. The values obtained significantly reduced with increasing levels of water restriction.

**Table 6.** Plasma osmolality of water-restricted Xhosa goats supplemented with VC

		Treatment								Probability		
Parameter	Day	W0	W70	W50	W70^+^	W50^+^	W70^++^	W50^++^	SEM	T	D	T × D
Na, mmol/L									1.21	**	***	ns
	30	135.33^b^	138.00^a^	139.00^a^	135.00^b^	137.00^b^	133.00^c^	134.67^c^				
	60	136.67^bc^	136.00^c^	138.67^ab^	136.67^bc^	139.67^a^	137.67^abc^	137.00^bc^				
	75	136.65^d^	138.00^cd^	142.00^a^	140.33^bc^	143.67^a^	137.67^d^	140.33^bc^				
K, mmol/L									0.16	**	***	***
	30	5.40^c^	5.70^b^	6.10^a^	5.43^c^	4.80^e^	5.13^d^	5.23^cd^				
	60	4.70^c^	5.00^b^	4.60^c^	5.00^b^	5.00^b^	5.13^ab^	5.33^a^				
	75	4.52^c^	5.06^b^	5.13^b^	5.06^b^	4.70^c^	4.47^c^	5.60^a^				
Mg, mmol/L									0.05	**	***	**
	30	0.95^a^	0.87^a^	0.65^c^	0.86^a^	0.74^b^	0.87^a^	0.66^c^				
	60	1.00^a^	0.78^b^	0.65^c^	0.90^a^	0.71^b^	0.93^a^	0.79^b^				
	75	1.19^a^	1.13^ab^	1.03^bc^	1.01^cd^	1.05^bc^	0.96^cd^	0.94^d^				
Cl, mmol/L									1.42	*	***	*
	30	100.67^bc^	103.33^ab^	105.00^a^	96.67^de^	95.33^e^	97.67^de^	100.33^ab^				
	60	100.00^b^	101.67^b^	104.67^a^	100.67^b^	96.00^c^	100.00^b^	95.00^c^				
	75	101.67^b^	105.00^b^	105.67^a^	101.27^b^	102.00^ab^	102.67^ab^	101.52^b^				
Ca, mmol/L									0.05	**	***	***
	30	2.05^a^	1.95^ab^	1.62^d^	1.86^b^	1.84^c^	1.94^abc^	1.86^b^				
	60	2.10^a^	1.85^b^	1.73^c^	1.86^b^	2.08^a^	2.08^a^	2.02^a^				
	75	2.12	2.10	2.02	2.07	2.11	2.12	2.05				

D, day effect; T, treatment effect; T × D, interaction between treatment and day; W, water restriction.

^abc^Means with different superscript down the column are significantly different (*P* < 0.05).

ns, *P* > 0.05; *P < 0.05; **P < 0.001; ***P < 0.0001.

^+^3 g VC daily; ^++^3 g VC daily + extra 5 g VC every eighth day.

**Table 7.** Blood chemistry of water-restricted Xhosa goats supplemented with VC

		Treatment								Probability		
Parameter	Day	W0	W70	W50	W70^+^	W50^+^	W70^++^	W50^++^	SEM	T	D	T × D
Urea, mmol/L									0.48	**	**	**
	30	5.10^d^	7.07^bc^	8.63^a^	6.85^c^	7.80^ab^	7.10^b^	8.25^a^				
	60	8.60^c^	8.73^c^	10.33^ab^	7.50^d^	9.73^b^	8.55^c^	11.00^a^				
	75	8.57^c^	11.27^a^	11.77^a^	9.30^bc^	11.43^a^	9.80^b^	11.73^a^				
Creatinine, mmol/L									3.26	**	**	*
	30	53.67	54.00	55.67	56.00	55.33	54.00	56.67				
	60	40.00^c^	43.33^bc^	49.33^ab^	48.33^ab^	50.00^a^	40.00^c^	45.00^abc^				
	75	43.00	45.00	48.67	44.00	48.00	43.00	46.67				
Glucose, mmol/L									0.12	**	**	**
	30	3.23^a^	2.80^b^	2.37^c^	2.97^b^	2.33^c^	2.40^c^	2.30^c^				
	60	2.47^a^	2.37^ab^	2.20^bc^	2.40^ab^	1.97^d^	2.42^a^	2.00^cd^				
	75	3.00^a^	2.90^ab^	2.70^bcd^	2.97^b^	2.53^de^	2.37^ab^	2.45^e^				
Total protein, g/L									2.20	**	**	**
	30	51.00^f^	60.67^cd^	70.00^a^	56.67^de^	65.67^ab^	53.67^ef^	63.33^bc^				
	60	52.00^c^	54.33^c^	73.67^a^	54.00^c^	63.33^b^	53.00^c^	65.00^b^				
	75	50.67^c^	68.00^ab^	68.00^ab^	65.67^b^	69.67^ab^	66.33^b^	71.67^a^				
Albumin, g/L									0.71	*	**	ns
	30	13.00^c^	14.00^abc^	15.33^a^	13.67^bc^	14.00^abc^	11.67^d^	14,67^ab^				
	60	12.00^b^	12.67	13.00^b^	12.00^b^	14.67^a^	12.33^b^	14.76^a^				
	75	14.67^d^	14.67	15.33^c^	16.00^ab^	15.00^cd^	15.67^bc^	16.67^a^				
Globulin, g/L									2.45	**	**	ns
	30	38.00^d^	46.67	48.66^bc^	45.00^c^	51.67^ab^	40.00^d^	56.00^a^				
	60	40.00^c^	41.66^c^	50.33^b^	42.00^c^	48.66^b^	40.67^c^	61.01^a^				
	75	36.00^d^	53.33^bc^	55.00^b^	49.65^c^	54.67^b^	50.66^bc^	63.33^a^				
Bilirubin, μmol/L									0.68	ns	ns	*
	30	6.67	7.00	7.67	7.00	7.33	7.00	7.33				
	60	5.33	6.67	7.00	6.00	6.33	7.00	7.00				
	75	5.33	7.33	7.33	6.33	7.67	6.33	6.00				
ALT, U/L									1.27	**	**	ns
	30	17.00	18.00	18.67	19.00	18.00	17.00	19.00				
	60	16.00^b^	16.33^b^	21.33^a^	16.33^b^	20.33^a^	19.00^a^	20.67^a^				
	75	18.33^b^	22.00^a^	22.26^a^	20.00^ab^	21.50^a^	21.00^ab^	22.07^a^				
ALP, U/L									1.54	**	**	**
	30	30.67^bc^	33.67^ab^	23.00^d^	32.00^bc^	29.00^c^	36.00^a^	25.00^d^				
	60	38.00^a^	38.33^a^	28.33^c^	39.33^a^	32.00^b^	40.00^a^	30.00^bc^				
	75	44.33^b^	46.00^ab^	37.67^c^	47.67^a^	40.67^c^	45.00^ab^	44.67^b^				
Cholesterol, mmol/L									0.15	ns	**	ns
	30	1.30	1.56	1.55	1.36	1.35	1.34	1.47				
	60	1.38	1.39	1.55	1.39	1.47	1.34	1.30				
	75	0.95^b^	1.11^ab^	1.34^a^	1.18^ab^	1.16^ab^	1.20^ab^	1.09^ab^				
HDL, mmol/L									0.08	*	*	ns
	30	0.80^b^	1.08^a^	0.95^ab^	1.03^ab^	0.95^ab^	0.83^b^	1.07^ab^				
	60	0.83	0.99	1.08	1.05	1.01	0.88	0.98				
	75	0.75	0.93	0.80	0.83	0.97	0.87	0.87				
LDL, mmol/L									0.06	**	**	**
	30	0.30^b^	0.42^ab^	0.47^a^	0.32^b^	0.37^ab^	0.30^b^	0.38^ab^				
	60	0.23^b^	0.37^a^	0.35^a^	0.32^ab^	0.35^a^	0.34^ab^	0.36^a^				
	75	0.12	0.13	0.20	0.16	0.25	0.14	0.23				
Triglyceride, mmol/L									0.04	**	*	ns
	30	0.18^bcd^	0.24^ab^	0.16^cd^	0.25^ab^	0.12^d^	0.26^a^	0.13^d^				
	60	0.17^cd^	0.25^ab^	0.17^cd^	0.28^a^	0.18^bcd^	0.26^ab^	0.12^d^				
	75	0.16^b^	0.29^a^	0.16^b^	0.27^a^	0.22^ab^	0.23^ab^	0.15^b^				

D, day effect; T, treatment effect; T × D, interaction between treatment and day; W, water restriction.

^abcd^Means with different superscript down the column are significantly different (*P* < 0.05).

ns, *P* > 0.05; *P < 0.05; **P < 0.001; ***P < 0.0001.

^+^3 g VC daily; ^++^3 g VC daily + extra 5 g VC every eighth day.

## DISCUSSION

### THI, DMI, and body weight changes

The THI experienced by the Xhosa ear-lobe goats was above the threshold values and this has resulted in severe stress, especially in the afternoon. This connotes that more heat was gained by the animal from the environment. When body thermal gradients (internal and external) are under the thermoneutral zone, heat is dissipated to the external environment. However, exposure of animals to extreme heat stress results in the flow of heat from the external environment to the animal’s body (Ames, 1980). The similarity in response observed in the animals’ skin’s temperature following water restriction suggests that the Xhosa ear-lobe goats can maintain their thermal balance load even at water restriction level of 50% ad libitum intake and during severe heat stress. Despite the combined stress of suboptimal WI and high thermal load, the total body thermal gradients across the experimental groups were similar. Recently, studies have suggested that ruminants exposed to extreme heat stress and under high thermal load adapt by constricting their thermal gradients between the environment and their bodies. This is made possible by the increased blood flow to the skin surface resulting from the elevated vasolidation of the skin capillaries induced by increased skin temperature (Katiyatiya et al., 2017). Limited WI resulted in body weight loss in this study. Similar results of weight loss resulting from water restriction levels have also been reported in other studies (Ghanem et al., 2008). Also, Alamer and Al-hozab (2004) reported 18% weight loss in Awassi sheep following 3 d of water deprivation during summer. Reduction in total body water and body solid loss during water deprivation is attributed to the usually observed body weight loss and can be exacerbated when the ambient temperature is very high. Also, the animal falls back to its body reserves due to reduced feed intake induced by suboptimal WI. A drop in the DMI as observed in this study also agrees with other studies (Casamassima et al., 2016). This adaptive nature to reduce feed intake following water restriction discourages the packing of feed in the digestive system. This may be due to the need to optimize food digestion with the minimal water that is below the required amount. All VC-treated groups tended to lessen the effect of weight loss when compared to the untreated water-restricted groups. A similar result of improved growth rate in weaned pigs, following ascorbic acid supplementation, has been reported (de Rodas et al., 1998). The WI:DMI ratio decreased as the level of water restriction increases. However, De Santos et al. (2019) reported an increase in WI:DMI ratio in animals that underwent water restriction for 24, 48, and 72 h. The observed differences could be due to the fact that the animals had ad libitum access to water every 24 h at the end of each water restrictions’ duration. The animals under the present study had their water, restricted for the entire duration of the trial. According to NRC (2007), 2.87 l of water will be consumed for each kilogram of DM ingested in an animal. This might explain the progressive decrease in the WI:DMI ratio as the water restriction increases.

### RcT and RR

In this study, the change in RcT remained within the reported range of 1.1 °C (Degen, 1977). This tendency of RcT to remain within a particular range has been reported for different environmental conditions (Lucena et al., 2013). Generally, an increase in RcT is often attributed to heat and/or water stress. However, water restriction effect was not significant on the RcT in this study and this agrees with other reports (Hamadeh et al., 2006). This implies that the Xhosa ear-lobe goat breed can adapt to the stressful conditions of limited WI. The mechanism by which VC reduces RcT is yet to be elucidated. The RR values recorded in this study decreased as the water restriction levels increased, especially in the water-restricted untreated groups. The RR of Lacauna ewes dropped when water restricted at both W80% and W60% of ad libitum water from day 0 to 14 (Casamassima et al., 2016). During the period of water scarcity and suboptimal WI, small ruminants adapt by reducing their respiratory activities in an attempt to curb the excessive loss of water and dehydration through pulmonary evaporation (Casamassima et al., 2016). Contrary to this study, Nejad et al., (2017) indicated an increase in the RR of Corriedale ewes, water deprived for 2 and 3 h compared to the control.

### FAMACHA and BCs

All the water-deprived experimental groups had small BCs compared to the water ad libitum group. Suboptimal WI affects feed intake, consequently leading to a loss in body condition (Sejian et al., 2010). The similarity in the BCs among the water-restricted groups indicates the ability of the goats to still maintain their body fat reserves. Supplementation of VC did not improve the BCs as compared to the water-restricted untreated groups. The FAMACHA system helps in identifying anemic goats and sheep by checking the ocular mucosa and comparing with a standard chat. By this assessment, anemic animals requiring urgent antihelminthic treatment can easily be identified (van Wyk and Bath, 2002). Accordingly, scores of 3, 4, and 5 in goats are indicative of anemia and require selective treatment (van Wyk and Bath, 2002). The FAMACHA scores recorded in this study were ≤3.33, representing a very minimal gastrointestinal nematode load. Despite the reduced feed intake and weight loss in the water-restricted groups, the low FAMACHA scores obtained, coupled with good housing care and management, indicate the ability of the goats to still maintain a good nutritional level. Supplementation of VC at single or multiple doses did not significantly reduce the FAMACAHA scores.

### Blood Metabolites

The increased plasma concentrations of urea, total protein, and ALT in response to water restriction levels observed in this study were lowered following VC treatment. A similar outcome was observed in VC-treated, water-deprived Awassi ewes (Ghanem et al., 2008). The welfare of animals can be assessed by serum concentration levels of ALT (together with aspartate transaminase) as a higher concentration of this enzyme in stressed goats is indicative of haemoconcentration and their adaptive capability (Banerjee et al., 2015). Values obtained for ALP in this study decreased with increasing levels of water restriction, with the W50 untreated group being the most affected. The serum enzyme ALP in an animal is often associated with metabolic activities. Chaidanya (2015) attributed the generally low enzyme levels in heat-stressed animals to a metabolic shift. Other studies, however, have reported that stress has no significant effect on the plasma ALP concentration (Pragna et al., 2018). The values obtained for serum concentration of albumin and protein decreases in water-restricted groups following VC supplementation and further decreases at multiple dosages. Contrarily, serum levels of albumin and protein in heat-stressed Japanese quails were reported to increase following VC supplementation (Sahin et al., 2003). The plasma glucose level in this study decreases as the water restriction levels increase and this could be attributed to the reduced availability of nutrients due to low feed intake. When Sudanese desert sheep were watered every 72 h, a 13% decline in plasma glucose level was reported (Hadjigeorgiou et al., 2000). However, some authors have reported that plasma glucose concentration remains unchanged in small ruminants following water restriction (Hamadeh, et al., 2006). Supplementation of single and/or multiple doses of VC to the animals in this study had no significant influence on the plasma glucose. Contrarily, the serum glucose in male rabbits was reported to decrease following VC supplementations (Yousef, 2004). The concentrations of cholesterol, HDL, and LDL in this study increased with levels of water restriction and their levels were not reduced in the VC-treated groups. Similar reports have been documented in water-restricted Yankasa ewes and Sudanese desert sheep (Hamadeh et al., 2006). Supplementation of VC to animals has been reported to lower cholesterol (Yousef, 2004). However, the supposed hypocholesterolemic effect of VC contravenes the documented report in this study. The need to meet the shortfall in dietary energy supply, an indication of fat mobilization, has been linked to the supposed increase in cholesterol following water restriction.

The increased sodium (Na^+^) concentration in this study agrees with the finding of Ghanem et al. (2008). An increase in aldosterone and vasopressin levels following water restriction usually results in increased renal retention and elevated Na^+^ concentrations (Ashour and Benlemlih, 2001). A similar trend is expected for chloride concentrations (Cl^−^), giving its passive distribution along the electrical gradients established by the active Na+ transport (Tasker, 1971). A sustained increase in the concentration of Na+ and Cl^−^ following limited WI may predispose an animal to salt poisoning as a result of increased accumulation of salt in the nervous tissue (Tasker, 1971). Both single and/or multiple doses of VC lowered the concentration of Na^+^ and Cl^−^ compared to the water-restricted untreated group. The increased blood potassium concentration following water restriction levels reported in this study contravenes the findings of Ghanem et al. (2008). Studies have also found that dehydration could either decrease blood K^+^ levels or not affect it (Hamadeh et al., 2006). A decrease in calcium and magnesium concentration following water restriction levels in this study may be attributed to the reduced dietary intake. VC supplementation, both at single and multiple doses, did not attenuate this decrease in concentration. The modulatory role of vitamin on electrolyte balance is not clear yet. Other studies on electrolyte balance following VC supplementation on Japanese quail and pigs have also been inconclusive (Avci et al., 2005).

## CONCLUSION

Limited WI in Xhosa goats under high ambient temperature revealed some remarkable changes. Goats had reduced body weight, DMI, RR, and BCs and increased blood metabolites. However, the body thermal gradients were not affected. Following VC supplementation, body weight loss, DMI depression, and higher blood concentrations were lessened. Contrary to our expectation, multiple VC dosing did not additively improve on the parameters where daily single dosing was positive.
